# HPTE-Induced Embryonic Thymocyte Death and Alteration of Differentiation Is Not Rescued by ERα or GPER Inhibition but Is Exacerbated by Concurrent TCR Signaling

**DOI:** 10.3390/ijms221810138

**Published:** 2021-09-20

**Authors:** Eddie Avellaneda, Atalie Lim, Sara Moeller, Jacqueline Marquez, Priscilla Escalante Cobb, Cristina Zambrano, Aaditya Patel, Victoria Sanchez, K. Godde, Christine Broussard

**Affiliations:** 1 Department of Biology, University of La Verne, La Verne, CA 91750, USA; eddie.avellaneda@laverne.edu (E.A.); atalie.lim@laverne.edu (A.L.); smoeller@stlucys.com (S.M.); jmarquez@llu.edu (J.M.); priscilla.escalante@laverne.edu (P.E.C.); aadi.r8@gmail.com (A.P.); vasanchez1992@gmail.com (V.S.); 2 Department of Pharmacology, School of Medicine, University of California San Diego, La Jolla, CA 92093, USA; cristina.zambrano@laverne.edu; 3 Department of Sociology/Anthropology, University of La Verne, La Verne, CA 91750, USA; kgodde@laverne.edu

**Keywords:** endocrine disrupting chemicals, estrogenic endocrine disruptors, HPTE, embryonic thymocytes, T cell development, cell death, thymocyte differentiation, estrogen receptor alpha (ERα), G protein-coupled estrogen receptor (GPER)

## Abstract

Organochlorine pesticides, such as DDT, methoxychlor, and their metabolites, have been characterized as endocrine disrupting chemicals (EDCs); suggesting that their modes of action involve interaction with or abrogation of endogenous endocrine function. This study examined whether embryonic thymocyte death and alteration of differentiation induced by the primary metabolite of methoxychlor, HPTE, rely upon estrogen receptor binding and concurrent T cell receptor signaling. Estrogen receptor inhibition of ERα or GPER did not rescue embryonic thymocyte death induced by HPTE or the model estrogen diethylstilbestrol (DES). Moreover, adverse effects induced by HPTE or DES were worsened by concurrent TCR and CD2 differentiation signaling, compared with EDC exposure post-signaling. Together, these data suggest that HPTE- and DES-induced adverse effects on embryonic thymocytes do not rely solely on ER alpha or GPER but may require both. These results also provide evidence of a potential collaborative signaling mechanism between TCR and estrogen receptors to mediate adverse effects on embryonic thymocytes, as well as highlight a window of sensitivity that modulates EDC exposure severity.

## 1. Introduction

Endocrine disrupting chemicals (EDCs) are environmental contaminants that perturb hormonal systems [1] by mimicking, blocking, or interfering with any aspect of hormone action in the body’s endocrine system [2,3]. Considering the ubiquity of EDCs in the built and natural environments, the fact that humans world-wide encounter these chemicals on a daily basis and have measurable levels of them in blood, fat, urine, and other tissues, and the potential long term effects disruption of development can have on health and disease, it is imperative to understand the role of EDC exposure in adverse health outcomes [1,2,4,5]. Recognition of the far-reaching implications of developmental endocrine disruption fomented the formation of a new field of study, the developmental origins of health and disease, concentrating early-life exposures and their consequences [6]. Insults during this period can lead to alterations in development that permanently affect function but may not be detected until later in life [1,7]. Of particular concern are the potential health impacts of developmental immunotoxicity [6,7,8]. Disruption of the developing immune system not only affects the immune system itself but also every other system of the body with which it interacts during development. Thus, developmental exposure to endocrine disrupting chemicals has been linked to a spectrum of adverse health outcomes [6].

The immune system evolved to mount protective responses against harmful pathogens or their biological products [9]. The immune system has two fundamentally different types of responses to pathogens: (1) the innate response, which occurs to the same extent however many times the infectious agent is encountered, and (2) the adaptive response, which improves upon repeated exposure to the same pathogen [10]. Adaptive immunity consists of antigen specific reactions mediated by stochastically re-arranged antigen receptors on T and B lymphocytes and is characterized by ‘memory’, which allows a much more vigorous and rapid immune response to be mounted upon subsequent exposure to pathogens [11]. The focus of this research is the developmental impact of EDCs on embryonic thymocytes, which give rise to the T lymphocytes that are a pivotal component of the adaptive immune response. Thymocyte development begins as committed lymphoid progenitors that arose in the bone marrow, migrate via the blood to the thymus, where they lose potential for B cell and natural killer cell development, resulting in a double negative (CD4−CD8−; DN) committed T cell precursor [12]. Once in the thymus, these cells will proliferate and undergo gene rearrangement to produce double positive (CD4+CD8+; DP) thymocytes [13]. Next, the population of DP thymocytes goes through positive selection, negative selection, and lineage-specific development to ensure that the surviving cells are optimized to function in a particular host environment [12,13]. More than 90% of thymocytes succumb to cell death by neglect, a failure of positive selection (because their rearranged T cell receptor is non-functional and/or cannot receive sufficient survival signals), or as a result of negative selection (excessive T cell receptor signaling), a form of central tolerance that rids the body of potentially autoreactive cells [13].

The thymus plays an integral part in the developmental process, primarily due to its facilitative role in thymocyte differentiation and maturation. Therefore, any disturbances of the thymus during the critical periods of pre-natal development can result in later life clinical disorders, such as autoimmune disorders [14,15] or aberrant infectious disease responses [16]. Previous studies have explored the impacts of various synthetic estrogenic chemicals on thymocyte development and shown that EDCs induce atrophy of the thymus by means of apoptosis or developmental blockade at particular stages of thymocyte development [17,18,19,20,21,22,23,24]. Studies have also reported synthetic-estrogen-induced alterations in the proportions of thymocyte subpopulations, especially DP thymocytes, which show the greatest declines in absolute cell counts [17,20,21,22,23,24,25,26]. Altogether, these studies illustrate the sensitive nature of thymocyte development, particularly during the prenatal period, where unperturbed hormone signaling and T cell receptor (TCR) expression and ligation are critical for appropriate immune system development and function. However, it remains unclear what mechanisms or pathways are being used to mediate the adverse effects of EDC exposure on thymocyte development.

It is clear that estrogen receptors are critical to normal mouse thymic development and are involved in (at least) estrogen-induced atrophy and alteration of development. A study by [27] using estrogen receptor alpha (ERα) knockout (ERKO) found that compared with wild type mice, male and female ERKO mice had smaller thymi with fewer cells. Studies of ERβ knockouts showed that while ERα expression is required for achieving a full-size thymus, ERβ is not [28]. Additionally, a study by Staples et al. [29] evaluated ERα participation in estradiol-induced thymic alterations in B6Ly5.1 mice. It showed that following estradiol treatment, ERKO mice lost 50% of their thymocytes, whereas wildtype mice lost 80% of them, indicating ERα is also involved in estrogen-induced thymocyte death. In sum, these studies support an important role for ERα in the development of the thymus and may play a role in estrogen and EDC adverse effects. However, another study by Staples et al. [30] indicated that ERα may only partially mediate the regulatory effects of estrogen, suggesting either another ER in the thymus/bone marrow or a separate receptor-independent pathway must be facilitating the partial thymic atrophy of ERα knockout mice. Studies of GPER/GPR30 suggest that it may be the alternative target receptor. Wang et al. [23] examined ERKO or GPR30 knockouts for thymocyte apoptosis and development blockades and showed that disruption of ERα or GPER/GPR30 lessened E2-induced development blockade or apoptosis of thymocytes. Furthermore, elimination of ERα resulted in a blockade of CD44+CD25− double negative thymocyte subpopulations, while elimination of GPER/GPR30 showed a preferential induction of apoptosis in TCRβ^−/low^ DP thymocytes. Thus, the cumulative results of these studies offer evidence for the role of ERα and GPER in the modulation of thymic development through estrogen signaling.

The purpose of this research was to determine what role ERα and GPER play in the death or alteration of differentiating thymocytes induced by the EDCs HPTE or DES and whether the TCR signaling events during thymocyte development have an impact on that role. To simulate the events of T cell development, cells were cultured in the presence, singly or in combination, of surface receptor antibodies (CD2 and TCR) that trigger the early steps of maturation and induce partial differentiation of thymocytes from the DP stage to the CD4^intermediate^ stage (CD4+CD8^low^; [31]). Prior work exposing differentiating thymocytes to HPTE or DES showed that thymocytes underwent cell death and preferential loss of DP and single positive (SP; CD4+CD8− or CD4−CD8+) populations [24]. In this study, pre-treatment with ICI 182,780 (an ERα antagonist), G15 (a GPER antagonist), or G1 (a GPER agonist) was used to probe whether each receptor is used by the EDCs to induce death or alteration of differentiation. This was also the first study to examine if signaling during differentiation cooperates with EDC signaling to mediate adverse effects. Two exposure scenarios were set up, one in which the EDCs were added concurrently with the differentiation signals and another in which EDCs were added after thymocytes had been signaled. We report on the findings and share a new perspective on the mechanism of action of EDCs on T cell development.

## 2. Results

### 2.1. Estrogen Receptor Usage Study

To examine whether the estrogen receptor binding capacity of the EDCs was responsible for the death and alterations in differentiation observed in our prior work [24], a receptor inhibition study was undertaken in conjunction with an in vitro differentiation assay. The results of the ANOVAs with total cell population as the dependent variable are presented in Table 1. The only condition values that were not significant at the Bonferroni *p*-value cutoff of 0.0125 were anti-BOTH, DMSO in ER alpha inhibition, and GPER ligation. The block variable was significant across the analyses (Table 1), indicating that experimental procedures introduced some variation into the results.

#### 2.1.1. ER Alpha Inhibition

**Figure 1:**
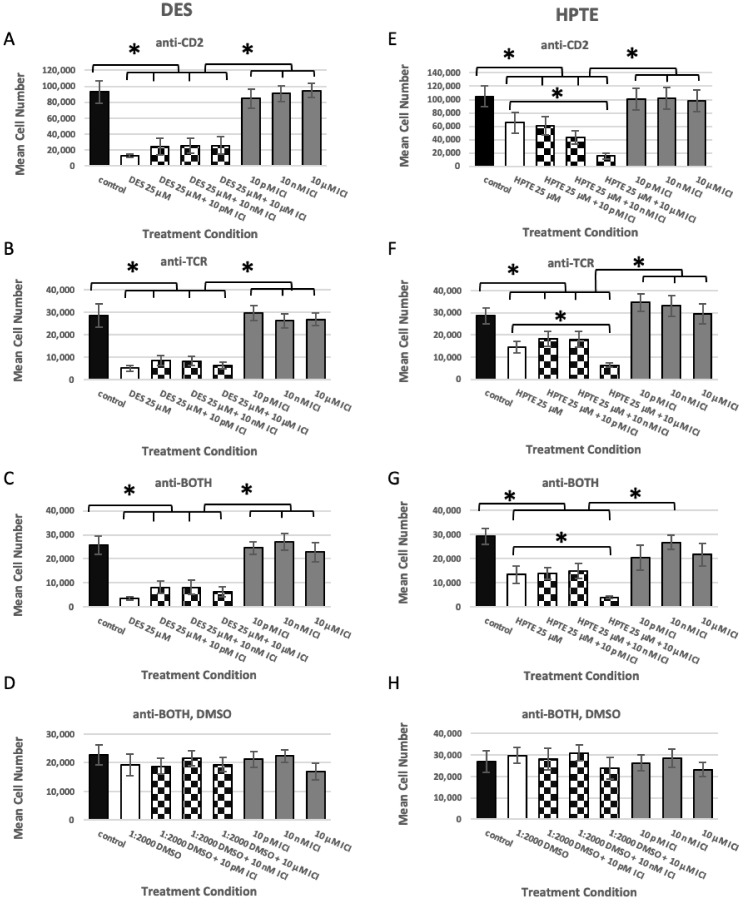
Estrogen receptor alpha inhibitor ICI 182,780 does not rescue DES-treated or HPTE-treated embryonic thymocytes from cell death. Embryonic thymocytes were treated with ICI prior to and during incubation in the differentiation culture. Pre-treated thymocytes were cultured 18–24 h in the presence of the antibody listed above each panel and 50 μM DES (**A**–**D**) or HPTE (**E**–**H**). DES N = 9, HPTE N = 8, * Tukey HSD post-hoc *p* < 0.05.

When embryonic thymocytes pre-treated with ICI 182,780 (ICI) were exposed to DES, the DES treatment still induced death (Figure 1A–C, checkerboard bars). It is interesting to note that the absolute number of live cells increased slightly compared with the EDC alone, but the difference between DES-treated (A–C, white bar) and DES plus ICI (A–C, checkerboard bars) was not statistically significant (*p* ≥ 0.05). Since DES by itself showed no significant difference from DES plus ICI at increasing concentrations, it appears that ICI did not rescue in vitro differentiated thymocytes from DES-induced death for any antibody condition. In vitro differentiated thymocytes treated with ICI by itself (Figure 1A–H, grey bars) also showed no significant difference from the medium control (Figure 1A–H, black bars), suggesting that ICI by itself did not significantly alter mean cell concentrations under any antibody conditions. In control experiments in which embryonic thymocytes were stimulated with anti-BOTH in the presence of only the organic solvent dimethyl sulfoxide (Figure 1D, anti-BOTH, DMSO), used to facilitate dissolving the EDCs in culture medium, cell numbers were not significantly different.

When embryonic thymocytes pre-treated with ICI were exposed to 25 µM HPTE (Figure 1E–G), the HPTE treatment also still induced death (Figure 1E–G, checkerboard bars). The cells were not rescued from HPTE-induced death, nor was there a trend of increasing cell numbers. In fact, the highest dose of ICI (10 µM, Figure 1E–G, bar 5, checkerboard) with HPTE resulted in a statistically significant reduction in cell number, compared with treatment with HPTE only. The exacerbation of cell death by ICI at the highest concentration when combined with HPTE was a trend not seen with 25 µM DES.

Embryonic thymocytes exposed to 25 µM HPTE (bar 2, white) or 25 µM HPTE plus 10 µM ICI (bar 5, checkerboard) were statistically significantly different under all antibody conditions (Figure 1E–G). However, whereas under the anti-CD2 (Figure 1E) or anti-TCR condition (Figure 1F), HPTE alone (bar 2, white) was significantly different from the medium control (bar 1, black) and from all three concentrations (bars 6–8, grey) of ICI alone; for the BOTH condition (Figure 1G) HPTE alone (bar 2, white) was only significantly different from the medium control (bar 1, black) and one of the ICI alone doses, 10 nM (bar 7, grey).

We also wished to determine whether ICI would selectively rescue any thymocyte subpopulations (DN, DP, CD4, CD8) involved in the EDC-induced death. However, none of the subpopulations were rescued by ICI pre-treatment (*p* ≥ 0.05).

#### 2.1.2. GPER Inhibition

**Figure 2:**
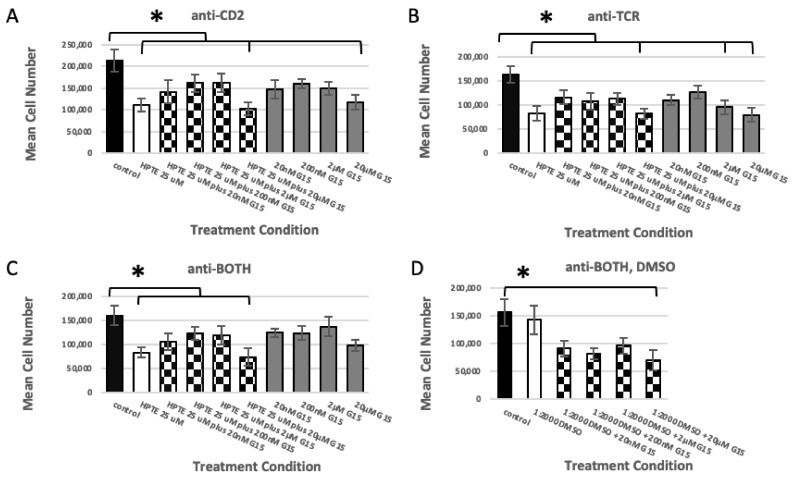
G protein-coupled estrogen receptor (GPER) inhibitor G15 does not rescue HPTE-treated embryonic thymocytes from cell death. Embryonic thymocytes were treated with G15 prior to and during incubation in the differentiation culture. Pre-treated thymocytes were cultured 18–24 h in the presence of the antibody listed above each panel and 25 μM HPTE (**A**–**D**). N = 8, * Tukey HSD post-hoc *p* ≤ 0.05.

To investigate whether the cell death induced by HPTE was due to its interaction with the nonclassical estrogen receptor GPER/GPR30, the selective antagonist G15 [32] was added to the in vitro differentiation assay. With the combination of HPTE and 20 nM, 200 nM, or 2 µM G15 (Figure 2A–C, bars 3–5, checkerboard), thymocyte death was reduced compared with HPTE by itself (Figure 2A–C, bar 2, white), but the change was not statistically significant (*p*_CD2_ = 0.9999; 0.6018; 0.6218; *p*_TCR_ = 0.2602; 0.7979; 0.2801; *p*_BOTH_ = 0.9964; 0.4604; 0.7626; respectively). The combination of 20 µM G15 and HPTE (Figure 2A–C, bar 6, checkerboard) consistently did not alter cell death compared with HPTE alone (Figure 2A–C, bar 2, white) under any antibody condition (*p*_CD2_ = 0.9996; *p*_TCR_ = 1.000; *p*_BOTH_ = 0.7660). Interestingly, similar to HPTE plus ICI at 10µM, addition of the GPER/GPR30 receptor antagonist by itself at 20 µM reduced viability of embryonic thymocytes compared with the control under all antibody conditions (Figure 2A–C, bar 10, grey and D, bar 6, checkerboard), but this reduction was only statistically significant under anti-CD2, anti-TCR, or BOTH/DMSO conditions (*p*_CD2_ = 0.0221; *p*_TCR_ = 0.0002; *p*_BOTH_ = 0.2168; *p*_BOTH/DMSO_ = 0.0066).

#### 2.1.3. GPER Ligation

**Figure 3:**
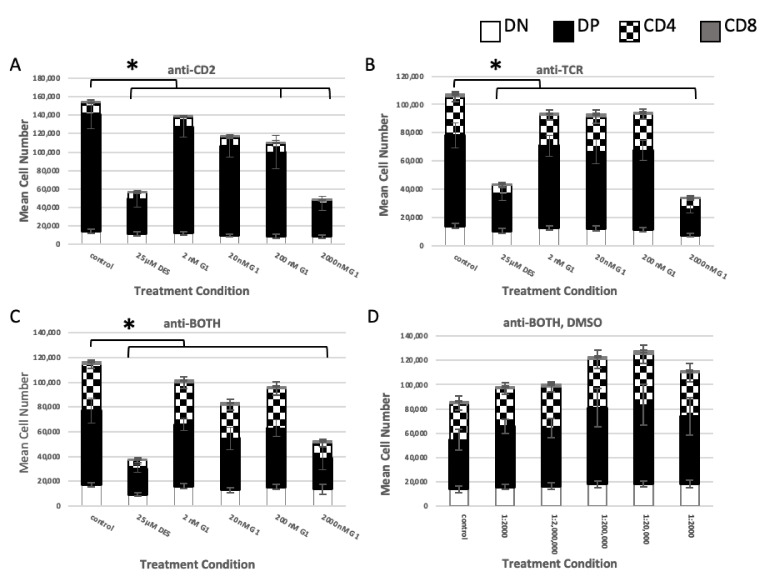
G protein-coupled estrogen receptor (GPER) agonist G1 induces cell death like DES in treated embryonic thymocytes. Embryonic thymocytes were treated with G1 and 50 μM DES during incubation in the differentiation culture. Treated thymocytes were cultured 18–24 h in the presence of (**A**) anti-CD2, (**B**) anti-TCR, (**C**) anti-BOTH, and (**D**) anti-BOTH, DMSO. N = 8, * Tukey HSD post-hoc *p* ≤ 0.05.

The reduction in death observed following G15 pre-treatment and exposure to HPTE, though not statistically significant, suggested that GPER may be involved in EDC-mediated death of embryonic thymocytes. To address whether activation of GPER would induce death, embryonic thymocytes were placed in the in vitro differentiation culture with the GPER agonist G1. As seen in Figure 3, at a dose of 2 µM (2000 nM, Figure 3), G1 reduced the mean live cell number under anti-CD2 (Figure 3A, bar 6), anti-TCR (Figure 3B, bar 6), and anti-BOTH (Figure 3C, bar 6) conditions. The observed decrease in cell number was statistically significant compared with the medium control (*p*_CD2_ = <0.0001; *p*_TCR_ = < 0.0001; *p*_BOTH_ = 0.0021) and comparable to embryonic thymocytes exposed to 25 µM DES under all antibody conditions (*p*_CD2_ = 0.9876; *p*_TCR_ = 0.5909; *p*_BOTH_ = 0.9898).

Prior work had shown that DN embryonic thymocyte numbers were not significantly decreased when subjected to DES exposure under anti-CD2 or anti-TCR conditions, whereas DP and CD4^intermediate^ cells were [24]. In the GPER agonist exposure experiment, DN mean cell numbers from G1-treated cultures were not statistically significantly different from DES-treated cultures under the anti-CD2, the anti-TCR, or the anti-BOTH conditions (Table 2, DN—rows 3, 6, 9) or from the medium control under the anti-CD2, the anti-BOTH, or the anti-BOTH, DMSO conditions (Table 2, DN—rows 2, 8, 11), but were statistically significantly different from medium control under the anti-TCR condition (Table 2, DN—row 5). These results suggested that DN cells were not consistently targeted by 2 µM G1.

As expected, 25 µM DES-treated DP mean cell number differences were statistically significant compared with the medium control under all antibody conditions (Table 2, DP—rows 1, 4, 7). DP mean cell numbers from 2 µM G1-treated cultures were also statistically significantly different from the medium control under all antibody conditions (Table 2, DP—rows, 2, 5, 8). However, under all antibody conditions, 25 µM DES-treated and 2 µM G1-treated mean cell numbers were not statistically significantly different (Table 2, DP—rows 3, 6, 9).

Generation of CD4^intermediate^ cells in the in vitro differentiation assay is dependent upon the combined presence of a CD2 and TCR signal. Therefore, fewer CD4^intermediate^ cells are generated under the anti-CD2 antibody condition due to the absence of the TCR signal (Figure 3A, checkerboard; [31]) and likely represent cells that were signaled prior to in vitro culture. However, a statistically significant reduction in mean cell numbers of CD4^intermediate^ was observed in cultures under the anti-CD2 antibody condition treated with G1 (Table 2, CD4^intermediate^—row 2), but not in cultures treated with 25 µM DES (Table 2, CD4^intermediate^—row 1). Moreover, mean cell numbers of CD4^intermediate^ in 25 µM DES-treated or 2 µM G1-treated were statistically significantly different under the anti-CD2 antibody condition (Table 2, CD4^intermediate^—row 3). CD4^intermediate^ mean cell numbers from 2 µM G1-treated cultures were not statistically significantly different from 25 µM DES-treated cultures under anti-TCR and anti-BOTH conditions (Table 2, CD4^intermediate^—row 6, 9), but were statistically significantly different from medium control (Table 2, CD4^intermediate^—row 5, 8).

The embryonic thymocytes were harvested between gestational days 16 and 18. Therefore, the majority of the cells in the CD8 subpopulation represented immature thymocytes prior to the DP stage and were small in number. CD8 mean cell numbers from 2 µM G1-treated cultures were not statistically significantly different from 25 µM DES-treated cultures under any antibody condition (Table 2, CD8—3, 6, 9), whereas both were statistically significantly different from the medium control, except for 25 µM DES-treated cultures under anti-CD2 conditions (Table 2, CD8—1, 2, 4, 5, 7, 8).

Thus, at 2 µM, G1 had effects similar to those of the exposure to 25 µM DES with regard to reducing mean cell numbers in the DP and CD4^intermediate^ subpopulations.

### 2.2. Role of TCR Signaling

EDCs mediate their actions through genomic or nongenomic mechanisms [33]. Possible mechanisms of action of DES or HPTE include nongenomic membrane-initiated steroid signaling [34,35,36,37,38]. Because signaling is also a critical aspect of thymocyte differentiation, we examined whether concurrent (added during signaling culture) or sequential (added during recovery culture) exposure to HPTE or DES changed the outcome of exposure.

#### 2.2.1. Concurrent versus Sequential Exposure

**Figure 4:**
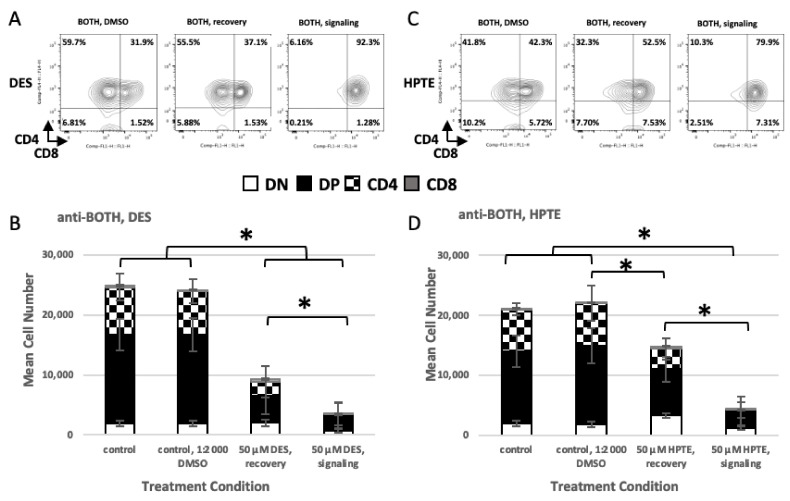
Concurrent TCR-signaling and EDC exposure worsens outcome for differentiating embryonic thymocytes. Embryonic thymocytes were treated with 50 μM DES (**A**,**B**) or HPTE (**B**) during the signaling (**A**,**C**—right panel or **B**,**D**—fourth bar) or recovery (**A**,**C**—middle panel, **B**,**D**—third bar) phases of the differentiation culture. Treated thymocytes were cultured 18–24 h in the presence of the antibody listed above each panel (**A**–**D**). DES N = 13, HPTE N = 9, * Tukey HSD post-hoc *p* < 0.05.

When the EDCs were added to signaling culture (Figure 4A, DES and B, HPTE, bar 4), a statistically significant loss of cell numbers was observed compared with the medium (Figure 4, bar 1; A, *p*_BOTH/signal_ = <0.0001; B, *p*_BOTH/signal_ = <0.0001) and diluent (Figure 4, bar 2; A, *p*_BOTH/signal_ = <0.0001; B, *p*_BOTH/signal_ = <0.0001) controls. When the EDCs were added to recovery culture (Figure 4A,B, bars 3), loss of cell numbers compared with medium and diluent controls was statistically significant for DES (Figure 4A, *p*_BOTH/recovery_ = <0.0001 and *p*_BOTH/recovery_ = <0.0001, respectively) and HPTE compared with the diluent control (Figure 4B, *p*_BOTH/recovery_ = 0.0497), but not for HPTE compared with the medium control (Figure 4B, *p*_BOTH/recovery_ = 0.0847). In addition, there was a statistically significant difference in cell numbers between cells that were exposed to EDCs during the signaling culture compared with cells exposed during the recovery culture (Figure 4A,B, compare bars 3 and 4; A, *p*_BOTH_ = <0.0001; B, *p*_BOTH_ = <0.0001). Fewer cells were lost when EDC exposure occurred in the recovery culture, i.e., after the cells had received the TCR signal. These results suggest that concurrent TCR signaling and EDC exposure led to maximal cell death, whereas prior TCR signaling was somewhat protective for subsequent exposure to DES or HPTE.

Examination of embryonic thymocyte subpopulations (DN, DP, CD4^intermediate^, and CD8) revealed differences in cell numbers. In DES-treated cultures, there was no statistically significant loss of DN thymocytes in cultures exposed during recovery phase compared with the medium and diluent controls (Table 3, DN comparisons, DES—rows 2, 4), whereas statistically significant loss of DN thymocytes was seen in cultures exposed during signaling phase compared with controls and with recovery phase exposed cultures (Table 3, DN comparisons, DES—rows 3, 5, 6). For HPTE, there were statistically significant changes in DN thymocyte cell numbers in cultures exposed during signaling phase and cultures exposed during recovery phase. However, DN thymocyte cell numbers decreased (as indicated by the negative difference value) in cultures exposed during signaling phase compared with controls (Table 3, DN comparisons, HPTE—rows 9, 11) and increased (as indicated by the positive difference value) in cultures exposed during recovery phase compared with controls (Table 3, DN comparisons, HPTE—rows 8, 10). Cultures exposed during signaling phase had decreased DN thymocyte cell numbers compared with cultures exposed during recovery phase (Table 3, DN comparisons, HPTE—row 12)

A statistically significant loss of DP thymocytes was seen in both exposure scenarios compared with controls (Table 3, DP comparisons, DES—rows 2, 3, 4, 5; HPTE—rows 8, 9, 11). Interestingly, differences between DP thymocytes from recovery HPTE-treated and medium control did not reach statistical significance (Table 3, DP comparisons, HPTE—row 10). Embryonic thymocytes that had received CD2 and TCR signals first, then EDC exposure in recovery culture, were resistant to EDC-induced cell loss, though the number of DP thymocytes still statistically significant decreased. However, fewer DP cells were lost when EDC exposure occurred in the recovery culture, a difference that was statistically significant (Table 3, DP comparisons, DES—row 6; HPTE—row 12).

A statistically significant loss of CD4^intermediate^ thymocytes was also seen in both exposure scenarios compared with controls (Table 3, CD4^intermediate^ comparisons, DES—rows 2, 3, 4, 5; HPTE—rows 8, 9, 10, 11). Fewer DP cells were lost when EDC exposure occurred in the recovery culture, a difference that was statistically significant (Table 3, CD4^intermediate^ comparisons, DES—row 6; HPTE—row 12).

CD8 thymocyte losses occurred in cultures exposed to DES during signaling phase compared with controls and to cultures exposed to DES during recovery phase (Table 3, CD8 comparisons, DES—row 3, 5, 6). There was no statistically significant change in CD8 thymocyte cell numbers in cultures exposed during recovery phase compared with the medium and diluent controls (Table 3, CD8 comparisons, DES—rows 2, 4). For HPTE, there were statistically significant changes in CD8 thymocyte cell numbers in cultures exposed during signaling phase compared with the diluent control and to cultures exposed during recovery phase, but not compared with the medium control (Table 3, CD8 comparisons, HPTE—rows 8, 11, 12). These inconsistencies in CD8 thymocyte cell numbers may be related to the mixture of immature and more mature cells in this pool and small cell numbers.

#### 2.2.2. Phenotypic Effects

**Figure 5:**
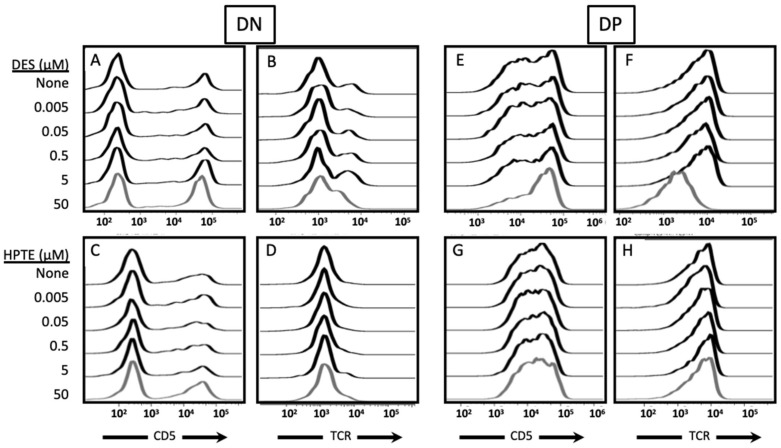
Embryonic thymocytes that survive transient exposure to DES or HPTE after exposure to differentiation signals have altered levels of TCR and CD5 on their surface. Cells were incubated in a two-day differentiation culture, with anti-CD2 and anti-TCR antibodies on day one. For recovery exposure, toxicants at concentrations indicated (0.005, 0.05, 0.5, 5, 50 µM) were added on day two after cells were washed and transferred to plates without the antibodies. Cells were stained at the end of the two-day incubation period with fluorochrome-conjugated antibodies against CD4, CD8, TCR, and CD5. Shown above are representative histograms from gated DN (**A**–**D**) or DP (**E**–**H**) embryonic thymocytes treated with the indicated concentrations of DES (**A**,**B**,**E**,**F**) or HPTE (**C**,**D**,**G**,**H**). Panels on the left are CD5 (**A**,**C**,**E**,**G**). Panels on the right are TCR (**B**,**D**,**F**,**H**). Recovery, DES N = 13, HPTE N = 9.

To further probe the differences in survival of embryonic thymocytes exposed to EDCs at different stages of in vitro differentiation culture, cells were stained post-culture for surface markers that identify subpopulations. Surviving DN and DP embryonic thymocytes exposed during signaling culture to 50 µM DES or HPTE exhibited reduced CD5 and reduced TCR expression on their surface compared with controls [24]. Surviving DN embryonic thymocytes exposed during recovery culture to 50 µM DES (Figure 5A,B—bottom trace) or 50 µM HPTE (Figure 5C,D—bottom trace) exhibited increased CD5 (Figure 5A,C; note the increased height of the CD5^high^ peak compared with top trace) and reduced TCR (Figure 5B,D; note the loss or leftward shift of the TCR^high^ peak compared with top trace) on their surface.

Surviving DP embryonic thymocytes exposed to 50 µM DES during recovery culture (Figure 5E,F—bottom trace) exhibited increased CD5^high^ expression (Figure 5E; note the increase in proportion of the population in the CD5^high^ peak compared with top trace) and reduced TCR expression (Figure 5F; note the leftward shift of the TCR^high^ peak compared with top trace) on their surface compared with controls (Figure 5E,F—top trace). Surviving DP embryonic thymocytes exposed to 50 µM HPTE during recovery culture appeared unchanged compared with controls (Figure 5G,H—compare bottom and top traces).

Therefore, exposure to EDCs in the recovery phase not only reduced the level of cell death, as seen in Figure 4, but also lessened the preferential loss of CD5^high^ and TCR^high^ cells in the DN and DP subpopulations.

## 3. Discussion

In our prior work [24], we showed that embryonic thymocytes undergoing differentiation in vitro died in response to exposure to the estrogenic endocrine disruptors HPTE or DES. The cell death observed also revealed an alteration of development by virtue of preferential loss of signaled (CD5^high^, TCR^high^) DP and CD4^intermediate^ thymocyte populations, key players in the selective processes of thymocyte development. In the current study, we aimed to elucidate whether the estrogen receptors ERα and GPER played an active role in EDC-induced adverse effects on differentiating embryonic thymocytes, and whether differentiation-associated signaling exacerbated these effects. Neither ICI 182,780 (ICI, ERα antagonist) nor G15 (GPER antagonist) rescued differentiating embryonic thymocytes. In fact, at the highest concentration used, ICI seemed to worsen cell death induced by HPTE. When ICI or G15 was added by itself to the differentiating thymocytes, neither ER inhibitor induced death on their own, except at the highest dose of G15 under some conditions. When G1 (GPER agonist) was added to differentiating embryonic thymocytes, similar declines in cell number and thymocyte subpopulations were observed compared with DES exposure. Lastly, this study was the first to demonstrate significant differences between differentiating embryonic thymocytes exposed to HPTE or DES during signaling phase versus recovery phase.

Our finding that G1 induces embryonic thymocyte death and subpopulation distribution alterations (i.e., preferential loss of differentiating DP and CD4^intermediate^ thymocytes) similar to that of DES, supports a role for GPER in EDC-induced adverse effects on thymocyte development. In addition, our observation that neither ER inhibitor (ICI or G15) by itself rescued adverse effects of HPTE or DES exposure on differentiating embryonic thymocyte supports the idea that ERα and GPER may be working in concert to trigger events in embryonic thymocytes leading to amplification of cell death and alteration of differentiation. Our results agree with ERKO and GPER studies [23,28,29,30] that established roles for both ERα and GPER, but not ERβ, in estrogen-mediated thymic effects. These studies showed that knockouts of either ERα or GPER alone did not completely abrogate thymocyte cell death in response to estrogen. Similarly, others have shown that ICI did not reverse the effects of HPTE or methoxychlor in ovarian toxicity [39].

Complicating our understanding of estrogenic endocrine disruptors is the fact that this category includes chemicals with a wide variety of mechanisms of action, including those that act directly or indirectly, activate or inhibit estrogen action, use genomic or non-genomic pathways, and potentially use more than one approach [36]. Genomic pathways can regulate target genes through direct estrogen receptor (ER)–DNA interactions or by associating with target DNA indirectly via tethering to other transcription factors [40]. Alternatively, non-genomic (rapid, signaling-based) pathways are mediated through membrane-associated ERs frequently associated with the activation of protein-kinase cascades, which may indirectly influence gene expression through signal transduction [41]. Studies of changes in gene expression in the thymus induced by estrogen [42], and a variety of chemicals that also induce thymic atrophy (including DES; [43]), revealed several functional categories of genes altered by chemicals with estrogenic activity. They included cell cycle, apoptosis, signal transducers, and development, categories critical for proper development of T cells. These findings support a role for changes in gene expression through estrogenic activity in altering T cell development, but do not address whether the accompanying mechanism is genomic or nongenomic.

Aside from the mechanisms of the receptors, the properties of the ER ligands also influence estrogenic activity. Chemicals such as HPTE and DES have been categorized by their known estrogenic properties [44], with the assumption that their mechanism of action would be due to their classical estrogenic activity. However, HPTE has been shown to act not only as an agonist of ERα but also an antagonist of ERβ [45] and to interact differently with ERα co-regulators than E2 and DES do [46]. DES has been shown to bind to [47,48] and be an agonist for both ERα and ERβ [49,50], and also to interact differently with ERα coregulators than E2 does [51]. In our hands, HPTE consistently showed weaker induction of cell death (50% reduction in thymocyte cell number) compared with DES (90% reduction in thymocyte cell number). More cells died with DES exposure than at the same concentration of HPTE, a difference that may not solely be explained by ER binding capacity. In addition, the interaction between HPTE and 10 µM ICI in our experiments revealed a synergistic effect on cell death that was not observed with DES. These results suggest variability in the mode of action of HPTE and DES.

Similarly, ER inhibitors have been shown to possess agonist activity in addition to their antagonist activity. ICI (an ERα antagonist) has been shown to have GPER agonist activity [52] that triggers ERK 1/2 activation through GPER/GPR30 [53]. In our study, ICI did not rescue HPTE or DES effects (Figure 1). Moreover, the exacerbation of death seen at the highest dose of ICI in our receptor inhibition study of HPTE action suggests that additional events are being triggered, perhaps through GPER/GPR30 ligation [52,54]. G15 (a GPER antagonist) has low-affinity cross-reactivity with ERα [55] with limited agonistic activity for gene expression changes, ~15% at 1 µM and 25% at 10 µM, and none for signaling changes [32]. In our study, blocking GPER/GPR30 with G15 did not appear to rescue HPTE-induced death; while mean cell numbers increased, the changes were not statistically significant. Conversely, 20 µM G15 kept mean cell numbers down in the presence or absence of HPTE, suggesting signaling independent of GPER. In addition, GPER inhibition with 20 µM G15 by itself resulted in statistically significant reductions in cell number when only anti-CD2 or anti-TCR were present and inconsistently with both (Figure 2), conditions that represent different combinations of signal transduction molecules associated with differentiation.

HPTE-induced apoptosis was observed as early as after 4 h in our prior work [24], a finding that suggests at least part of the mechanism used by HPTE is through signaling pathways. Three groups of membrane ERs have been identified: classical Erα and Erβ [56], isoforms of classical Ers [57,58], and GPER [59,60]. GPER is a 7-membrane-spanning protein with high affinity to E_2,_ thus binding of estrogen to GPER induces rapid non-genomic signaling [36]. Non-genomic pathways can also be triggered by direct interaction (crosstalk) between different membrane ER signaling pathways [52], crosstalk between signaling components of membrane ERs and other non-ER signaling pathways [60], and collaboration between ER signaling and growth factor, hormone, or another receptor activity [36,50]. Membrane-associated ER have been shown to activate signaling molecules (e.g., calcium, PI-3K, and small G proteins) and to associate with scaffold proteins (e.g., SHC), leading to the creation of signalosomes that trigger downstream signaling events, such as the activation of the MAPK and AKT pathways [38,59,61]. GPER has been shown to activate ERK1/2 [53] through the transactivation of EGFRs. G15 ligation of GPER has been shown to decrease MAPK, AKT, and cell-cycle proteins and to increase pro-apoptotic proteins, changes that result in cell-cycle arrest and apoptosis in human squamous carcinoma cells [62]. Another study demonstrated that GPER/GPR30 ligation also induces PI3K activation [59]. Recent research has suggested a variety of mechanisms that GPER may utilize in affecting estrogen signaling, such as GPER acting independently of ERα, GPER forming a complex with ERα or activating ERα, and, lastly, GPER and ERα signaling influencing parallel signaling pathways, which achieve the same cellular response [52].

Signaling through the T cell receptor utilizes many of the same signal transduction molecules observed to be modulated by membrane ER ligation [63]. Moreover, signal transduction intensity determines thymocyte fate in T cell development [13,64]. Thymocytes with nonfunctional T cell receptors or those that encounter low affinity peptides die by neglect, unable to receive survival signals. Thymocytes that receive moderate intensity TCR signals are positively selected, able to receive survival signals and differentiate. Thymocytes that receive very strong intensity TCR signals also tend to die, victims of negative selection/central tolerance. This range of TCR signal intensities and outcomes can be mimicked in vitro by stimulation of thymocytes with antibodies to various surface markers. Moderate intensity survival and differentiation signals can be provided by anti-TCR plus antibodies to number of different surface markers including CD2 [31] and high intensity death-inducing signal by anti-TCR and anti-CD28 antibodies [65]. Our results showed EDC exposure concurrent with differentiation signaling (anti-TCR plus anti-CD2) resulted in greater death than exposure after embryonic thymocytes had received differentiation signals. Thus, addition of the EDC at the same time as cells were receiving the differentiation signal had the effect of turning a moderate survival and differentiation signal into a high intensity death signal. These findings suggest that EDC exposure can exacerbate the effects of TCR signaling and vice versa. Taken together, our data and those of Brown and coworkers [25], obtained using high affinity H-Y TCR, suggest that thymocytes undergoing differentiation signaling may be more susceptible to EDC-induced death. Conversely, our findings that exposure to EDCs after differentiation signaling has less of an impact (less cell death) on developing thymocytes suggest that embryonic thymocytes that have already received the TCR signal are somewhat resistant to EDC effects. This interpretation is also supported by phenotypic analyses that showed that although some CD4+ intermediate and double positive subpopulations still died when EDCs were added post-differentiation signaling, more CD5^high^, TCR^high^ cells appeared to survive. Moreover, it has been observed that signaling through TCR and CD2 results in upregulation of expression of Bcl-2, an anti-apoptotic protein, in the in vitro differentiation assay [31], and Bcl-2 transgene expression partially rescues DES effects in vivo [66]. These results support the idea that TCR/CD2 signaling prior to EDC exposure is partially protective.

In conclusion, this study indicates that, while ERα or GPER may be critical to thymocyte development and EDC exposure, they do not appear to be acting alone. To further test this hypothesis, it will be informative to use ERα and GPER inhibitors together in the differentiation assay to determine whether in combination, they can abrogate HPTE or DES adverse effects. Additionally, our study is the first to demonstrate that signaling through estrogen receptors appears to work in concert with differentiation signaling to exacerbate EDC effects, perhaps mimicking the established collaborations between membrane ER and other non-ER receptors observed in other cell types [35,36,50]. Future studies using inhibitors to unique or shared signaling molecules may shed light on whether cross talk between the differentiation signaling pathways and EDC-induced signaling is responsible for EDC action and the exacerbation of EDC effects we saw when differentiation signals were concurrently provided to differentiating embryonic thymocytes. Finally, the results of our study also highlight the extreme sensitivity (regardless of mechanism) of differentiating embryonic thymocytes. Because differentiation and development of embryonic thymocytes represents the seeding of the immune system [67,68], this work underscores the importance of that window of development. Lesions from environmental exposures during embryonic development can have profound effects on the functioning of the immune system over the lifetime of the organism. Therefore, understanding how EDCs alter this process is critical work.

## 4. Materials and Methods

### 4.1. Animals

All experiments were approved and conducted under the supervision of the Institution’s Animal Care and Use Committee (IACUC; protocol code LV0002, 8 July 2009; LV0004, 12 May 2014; LV0005, 3 May 2017) of the University of La Verne in accordance with federal guidelines for the care and use of live animals in research [69]. C57BL/6 mice were purchased from Simonsen Labs (Gilroy, CA, USA). Timed matings were performed in house or by the vendor to produce embryos aged 16–18 days post-fertilization (dpf). Mice were maintained in polypropylene cages on a 12 h light cycle with food and water ad libitum.

### 4.2. Abs and Flow Cytometry Staining, Gating, and Acquisition

The following antibodies (BioLegend San Diego, CA, USA) were titrated and used to stain freshly isolated and cultured thymocytes: APC anti-CD4 (clone RM4-5, Rat IgG2a), FITC anti-CD8 (clone 53-6.7, Rat IgG2a), PE anti-CD5 (clone 53-7.3, Rat IgG2a), anti-T cell receptor (clone H57-597, Armenian Hamster IgG). Cells were stained with fluorochrome-conjugated antibodies for 30 min then washed with PBS containing 1% FBS.

A minimum of 10,000 events were collected per sample on the Accuri C6 system (BD Biosciences). Flow cytometry data files were analyzed using the FlowJo (TreeStar Inc., Ashland, OR, USA) software. Live gates were set on FSC vs. SSC plots and thymocyte population quad gates determined using single stains and FMO controls. Mean live cell numbers were determined by FlowJo software based on gating parameters.

### 4.3. In Vitro Differentiation Assay

All 96-well plates were prepared and incubated 24 h at 4 °C with purified antibodies (BioLegend San Diego, CA, USA) against CD2 (RM2-5, Rat IgG 2b) and the T cell receptor (TCR; H57-597, Armenian Hamster IgG), alone or in combination. The differentiation assay has two stages: signaling culture (stimulation of antigen receptor and/or adhesion molecule by antibodies) and recovery culture (rest period to allow re-expression of surface markers down-regulated due to signaling events). The combination of antibodies has been shown to induce differentiation of DP cells into CD4^intermediate^8^low^ thymocytes, a post-positive selection intermediate population in T cell development [31]. Embryonic thymocytes were harvested from pooled litters of gestational day (GD) 16–18 embryos. Therefore, each N represents an independent repeat with pooled thymi as the source of the embryonic thymocytes. Embryonic thymocytes (gd 16–18) were teased into single cell suspension in complete RPMI 1640 (supplemented with 10% fetal bovine serum, l-glutamine, 50 mM beta-mercaptoethanol, 1% penicillin/streptomycin), and 4.0 × 10^5^ cells were added per well. Thymocytes were cultured in the absence of phenol red to avoid confounding effects of phenol red estrogenicity [70]. Thymocytes were then incubated 18–24 h in the antibody-coated plates (signaling culture). At the end of the signal culture period, cells were removed by careful pipetting, washed in cRPMI, and transferred to a fresh, uncoated 96-well plate. Embryonic thymocytes were incubated an additional 18–24 h in the absence of antibodies (recovery culture) to facilitate re-expression of surface markers, such as the TCR, and completion of the differentiation process.

In order to determine whether DES or HPTE mediated their induction of death by nongenomic amplification of antigen receptor signaling or some other mechanism related to the TCR signaling during differentiation, we set up two parallel in vitro differentiation assays. The original exposure regimen called for the addition of the EDC during the signaling culture, such that TCR signaling and EDC exposure were concurrent. The second regimen entailed adding the EDC to the recovery culture, after the embryonic thymocytes had been removed from the antibody-coated plates and washed.

### 4.4. Chemicals and Treatment

Endocrine disruptors, diethylstilbestrol (DES; purity 100%), and HPTE (2,2-bis(p-hydroxyphenyl)-1,1,1-trichloroethane, purity 97%; metabolite of methoxychlor) were purchased from Sigma–Aldrich Chemical (Darmstadt, Germany). MXC is normally metabolized in the liver [71] to form (2,2-bis(p-hydroxyphenyl)-1,1,1-trichloroethane (HPTE) and 2-(p-hydroxyphenyl)-2-(p-methoxyphenyl) 1,1,1-trichloroethane. For the experiments presented, the metabolite HPTE was used instead of the parent molecule to avoid confounding effects of the lack of metabolism in primary culture. G1 (GPR30 agonist; [72]) was purchased from Tocris (Bristol, UK). Chemicals were added to in vitro differentiation assays during the signaling culture (DES, HPTE, or G1) or the recovery culture (DES or HPTE). For dose response studies, the concentrations of each toxicant used were 0.005, 0.05, 0.5, 5, and 50 µM. For single dose experiments, 25 or 50 µM DES or HPTE was used.

### 4.5. Receptor Inhibition Studies

ICI 182,780 is high affinity estrogen receptor antagonist (IC_50_ = 0.29 nM), shown to lack any partial agonism both in vitro and in vivo. It is also a high affinity agonist of the membrane estrogen receptor GPER/GPR30. For estrogen receptor alpha inhibition experiments, the inhibitor, ICI 182,780 (Sigma Aldrich, St. Louis, MO, USA), was added at various concentrations (10 pM, 10 nM, 10 mM) to thymocyte-seeded signaling cultures and incubated for one hour prior to EDC addition at 5% CO_2_, 37 °C, and 96% humidity. Pre-treatment was performed to ensure ICI blocking efficiency. After one hour, the EDCs were added and thymocytes incubated between 18 and 24 h in the signaling culture. Thymocytes were then washed and transferred to recovery culture.

G15 is a high affinity and selective GPER/GPR30 receptor antagonist (K_i_ = 20 nM). At ≥10 μM, G15 has affinity for Erα and Erβ. For GPR30 inhibition, the cells were placed in a 12-well plate and treated with various concentrations (20 nM, 200 nM, 2 mM, and 20 mM) of the GPR30 inhibitor G15 (EMD Biosciences, San Diego, CA, USA) or cRPMI and incubated for one hour at 37 °C, 5% CO_2_, and 95% humidity. Pre-treatment was performed to ensure G15 blocking efficiency. After exposure, the cells were transferred to the signaling culture and EDCs or control solutions were added. The plate was incubated between 18 and 24 h in the signaling culture. Thymocytes were then washed and transferred to recovery culture.

### 4.6. Statistical Analyses

Each experiment was subjected to a randomized complete block design two-way analysis of variance (ANOVA). The identification of independent (explanatory)/dependent (response) variables, blocked design, and most assumptions were applied similarly across analyses. The dependent variable (cell population: DN, DP, CD4, CD8, or total cell population), which represented the outcome of the experiment, was log transformed when it did not follow a normal distribution. The first independent variable was either the antibody condition (referred to as condition) or the experimental treatment (referred to as treatment). The second independent variable is a block designed to control for variation introduced by treating the cell populations at different times, different lengths of gestation (range was 16–18 days), and any other differences arising during treatment. Tukey Honest Significant Difference (HSD) post hoc tests, with adjustments for multiple comparisons, were performed to identify what groups were different from others by comparing the conditions/treatments to one another within each experiment.

A Bonferroni correction [73,74] was applied to the *p*-value cut off for some ANOVA tests in order to reduce the chance of a Type I error. The Bonferroni corrected *p*-value cutoff used for ER alpha inhibition, GPER inhibition, and GPER ligation experiments is 0.0125 (α = 0.05/4 cell populations), while the concurrent versus sequential exposure experiment did not require a correction (α = 0.05). The *p*-values for Tukey HSD post hocs were evaluated at α = 0.05 as the *p*-values reported are adjusted for multiple comparisons. All analyses were completed in R [75] and graphs were produced in Microsoft Excel.

## Figures and Tables

**Table 1:** Blocked 2-way ANOVA with total cell counts across treatments and conditions.

Experiment	Endocrine Disruptor	Condition	F-Value Treatment	*p*-Value Treatment	F-Value Experiment Number	*p*-Value Experiment Number
ER Alpha Inhibition						
	DES					
		anti-CD2	19.68	**<0.0001**	7.59	**<0.0001**
		anti-TCR	15.13	**<0.0001**	6.86	**<0.0001**
		anti-BOTH	20.76	**<0.0001**	4.95	**0.0001**
		anti-BOTH, DMSO	1.32	0.264827	13.92	**<0.0001**
	HPTE					
		anti-CD2	16.08	**<0.0001**	19.14	**<0.0001**
		anti-TCR	32.03	**<0.0001**	15.23	**<0.0001**
		anti-BOTH	13.13	**<0.0001**	6.15	**<0.0001**
		anti-BOTH, DMSO	0.95	0.480282	3.28	**0.008185**
GPER Inhibition						
	HPTE					
		anti-CD2	3.387	**0.00163**	8.398	**<0.0001**
		anti-TCR	5.106	**<0.0001**	8.783	**<0.0001**
		anti-BOTH	4.637	**<0.0001**	5.619	**<0.0001**
		anti-BOTH, DMSO	3.372	**0.0018**	11.123	**<0.0001**
GPER Ligation						
	DES					
		anti-CD2 *	18.433	**<0.0001**	8.551	**<0.0001**
		anti-TCR	18.234	**<0.0001**	7.168	**<0.0001**
		anti-BOTH	8.311	**<0.0001**	2.951	**0.0154**
		anti-BOTH, DMSO	1.201	0.329158	5.067	**0.000482**
Role of TCR Signaling						
	DES					
		anti-BOTH	110.83	**<0.0001**	19.52	**<0.0001**
	HPTE					
		anti-BOTH	56.442	**<0.0001**	6.984	**0.000112**

**Bold** indicates significant at Bonferroni corrected α = 0.0125. * Normal distribution, so not log transformed.

**Table 2:** Pairwise comparisons of thymocyte subpopulations from Tukey HSD post hoc tests.

	DN		DP		CD4 Intermediate		CD8	
Comparison	Difference/F-Value	*p*-Value	Difference/ F-Value	*p*-Value	Difference/ F-Value	*p*-Value	Difference/F-Value	*p*-Value
CD2								
ANOVA	2.62 (5.31)	0.0409 (<0.0001)	14.46 (7.05)	*<0.0001* (<0.0001)	9.98 (9.84)	*<0.0001* (<0.0001)	3.75 (8.35)	*0.0080* (<0.0001)
25 µM DES—medium	**	**	−1.2916	**0.0001**	−0.6251	0.1424	−0.1043	0.9973
2 µM G1—medium	**	**	−1.4876	**<0.0001**	−1.4826	**<0.0001**	−0.6912	**0.0492**
2 µM G1—25 µM DES	**	**	−0.1960	0.9623	−0.8575	**0.0161**	−0.5869	0.1335
TCR								
ANOVA	3.55 (4.61)	*0.0106* (0.001)	20.64 (9.06)	*<0.0001* (<0.0001)	13.31 (3.55)	*<0.0001* (0.0055)	6.27 (17.27)	*0.0003* (<0.0001)
25 µM DES—medium	−0.3080	0.4260	−0.8942	**<0.0001**	−1.4969	**<0.0001**	−0.7549	**0.0148**
2 µM G1—medium	−0.6148	**0.0074**	−1.2401	**<0.0001**	−1.3609	**0.0001**	−0.8667	**0.0036**
2 µM G1—25 µM DES	−0.3068	0.4305	−0.3459	0.2945	0.1359	0.9948	−0.1118	0.9950
BOTH								
ANOVA	2.02 (2.37)	0.1001 (0.0428)	7.62 (4.01)	*<0.0001* (<0.0001)	16.22 (3.3)	*<0.0001* (0.0085)	8.48 (14.95)	*<0.0001* (<0.0001)
25 µM DES—medium	**	**	−1.0104	**0.0020**	−1.8813	**<0.0001**	−1.2053	**0.0002**
2 µM G1—medium	**	**	−1.0655	**0.0010**	−1.2553	**0.0004**	−0.7800	**0.0274**
2 µM G1—25 µM DES	**	**	−0.0551	0.9999	0.6260	0.1887	0.4253	0.4917
BOTH, DMSO **								
ANOVA	0.84 (4.89)	0.5316 (0.0006)	1.37 (8.46)	0.261 (<0.0001)	1.1 (6.18)	0.378 (<0.0001)	1.03 (17.64)	0.418 (<0.0001)
diluent ^a^—medium	**	**	**	**	**	**	**	**
2 µM G1—medium	**	**	**	**	**	**	**	**
2 µM G1—diluent	**	**	**	**	**	**	**	**

*Italics* indicates Bonferroni corrected significance at 0.0125; Values in parentheses are from block variables. **Bold** indicates Bonferroni corrected significance at 0.05. ^a^ 1:2000 dmso. ** ANOVA tests not significant, thus post hocs not generated.

**Table 3:** Pairwise comparisons of thymocyte subpopulations exposed in recovery or signaling culture from Tukey HSD post hoc tests.

	DN		DP		CD4 Intermediate		CD8	
Comparison	Difference/F-Value	*p*-Value	Difference/F-Value	*p*-Value	Difference/F-Value	*p*-Value	Difference/F-Value	*p*-Value
*DES*								
ANOVA	30.61 (23.02)	*<0.0001* (<0.0001)	70.02 (11.96)	*<0.0001* (<0.0001)	76.09 (9.43)	*<0.0001* (<0.0001)	27.79 (19.17)	*<0.0001* (<0.0001)
medium—diluent ^a^	0.0335	0.9941	0.0009	0.9999	0.0414	0.9988	−0.1504	0.9090
recovery 50 µM—diluent	0.1305	0.7559	−1.2581	**<0.0001**	−1.2267	**0.0005**	−0.0105	0.9999
signal 50 µM—diluent	−0.9708	**<0.0001**	−1.9822	**<0.0001**	−3.7025	**<0.0001**	−1.8412	**<0.0001**
recovery 50 µM—medium	0.0970	0.8820	−1.2590	**<0.0001**	−1.2680	**0.0003**	0.1398	0.9251
signal 50 µM—medium	−1.0044	**<0.0001**	−1.9831	**<0.0001**	−3.7439	**<0.0001**	−1.6908	**<0.0001**
signal 50 µM—recovery 50 µM	−1.1014	**<0.0001**	−0.7241	**0.0006**	−2.4759	**<0.0001**	−1.8306	**<0.0001**
*HPTE*								
ANOVA	14.10 (11.01)	*<0.0001* (<0.0001)	27.86 (8.51)	*<0.0001* (<0.0001)	121.47 (5.23)	*<0.0001* (0.0008)	7.19 (4.74)	*0.0014* (0.0016)
medium—diluent	0.0294	0.9982	−0.0690	0.9778	0.0147	0.9999	0.1267	0.9839
recovery 50 µM—diluent	0.5518	**0.0193**	−0.5056	**0.0350**	−0.8879	**0.0045**	0.9787	0.0502
signal 50 µM—diluent	−0.6033	**0.0124**	−1.4530	**<0.0001**	−3.9733	**<0.0001**	−0.7007	0.2472
recovery 50 µM—medium	0.5223	**0.0283**	−0.4367	0.0810	−0.9026	**0.0039**	0.8520	0.1037
signal 50 µM—medium	−0.6327	**0.0084**	−1.3840	**<0.0001**	−3.9880	**<0.0001**	−0.8274	0.1351
signal 50 µM—recovery 50 µM	−1.1551	**<0.0001**	−0.9474	**0.0001**	−3.0854	**<0.0001**	−1.6794	**0.0007**

*Italics* indicates Bonferroni corrected significance at 0.0125; Values in parentheses are from block variables. **Bold** indicates Bonferroni corrected significance at 0.05. ^a^ 1:2000 dmso.

## Data Availability

The data presented in this study are available on request from the corresponding author.
